# A Portable Smartphone-Based System for the Detection of Blood Calcium Using Ratiometric Fluorescent Probes

**DOI:** 10.3390/bios12110917

**Published:** 2022-10-24

**Authors:** Yue Wu, Yunshan Zhang, Zhongyuan Xu, Xinyu Guo, Wenjian Yang, Xiaoyu Zhang, Yuheng Liao, Minzhi Fan, Diming Zhang

**Affiliations:** Zhejiang Laboratory, Research Center for Intelligent Sensing Systems, Hangzhou 311121, China

**Keywords:** hypocalcemia, calcium ions, smartphone, ratiometric fluorescence probe, bovine serum

## Abstract

Hypocalcemia is a disease that adversely affects the production and reproduction of dairy cows. A portable device for rapid bovine blood calcium sensing has been growing in demand. Herein, we report a smartphone-based ratiometric fluorescence probe (SRFP) platform as a new way to detect and quantify calcium ions (Ca^2+^) in blood serum. Specifically, we employed a cost-effective and portable smartphone-based platform coupled with customized software that evaluates the response of Ca^2+^ ions to ratiometric fluorescence probe in bovine serum. The platform consists of a three-dimensional (3D) printed housing and low-cost optical components that excite fluorescent probe and selectively transmit fluorescence emissions to smartphones. The customized software is equipped with a calibration model to quantify the acquired fluorescence images and quantify the concentration of Ca^2+^ ions. The ratio of the green channel to the red channel bears a highly reproducible relationship with Ca^2+^ ions concentration from 10 μM to 40 μM in bovine serum. Our detection system has a limit of detection (LOD) of 1.8 μM in bovine serum samples and the recoveries of real samples ranged from 92.8% to 110.1%, with relative standard deviation (RSD) ranging from 1.72% to 4.89%. The low-cost SRFP platform has the potential to enable campesino to rapidly detect Ca^2+^ ions content in bovine serum on-demand in any environmental setting.

## 1. Introduction

Hypocalcemia, a common nutritional metabolic disease, occurs in half of the multiparous cows and a quarter of the primiparous cows in intensive dairy farms in China [1]. A substantial amount of blood calcium is needed to synthesize milk after dairy cows calving, which leads to hypocalcemia in the immediate postpartum period. Subclinical hypocalcemia is easily overlooked by farms due to the lack of practical, rapid, and inexpensive field detection techniques. The blood calcium of dairy cows needs to be detected in real time, and the sample size of the detection is significant. Therefore, rapid and cost-effective detection of the calcium ions (Ca^2+^) concentration in cow serum is very useful for the diagnosis of subclinical hypocalcemia [2].

In recent decades, various methods including spectroscopic techniques [3], electrochemical method [4,5], inductively coupled plasma-atomic emission spectrometry (ICP-AES) [6,7], liquid chromatography [8], ion-selective electrodes [9], and atomic absorption spectroscopy (AAS) [10] have been used in the determination detection of Ca^2+^. Although these analytical instruments have excellent accuracy and usability in challenging detection scenarios, the time-consuming operation, complex sample preparation process, and high operating cost limit their application in the field of real-time rapid detection of blood calcium concentration in cow farms. With considerable critical attention to hypocalcemia in cows, it is highly desirable to develop a rapid, efficient, and sensitive assay for the point-of-care testing (POCT) of bovine blood calcium with simple operation.

Smartphones have been integrated as sensors for biological sciences and biological medicine detection due to their portability and unique availability, for instance, intelligent sensor chips [11,12] and intelligent detectors [13,14]. Real-time, point-based, and easy-to-use monitoring using high-resolution cameras and system software modules in smartphones as analysis units or control units can greatly simplify inspection system complexity and reduce design requirements. For example, Tang et al. provided a smartphone-based platform for the real-time detection of Ag^+^ in lake water. Based on the lyceum ruthenium derived carbon dots, the smartphone platform with the color (red–green–blue, RGB) analysis software were used to capture and analyze fluorescence images, accomplishing sensitive and reliable detection of Ag^+^ [15]. Xiao et al. developed a smartphone-based sensing system combined with a custom design App for rapid and easy detection of Hg^+^, Pb^2+^, and Cu^2+^ in the Pearl River water samples, showing satisfactory analytical performance [16]. These smartphone-based quantitation systems are greatly promoting the progress of intelligent detection technology [17,18,19]. 

The method of fluorescence detection in POCT has the advantages of good reproducibility, fast reaction time, low cost, on-site monitoring, and strong applicability. The main detection mechanisms can be roughly classified into three categories: fluorescence quenching, fluorescence enhancement, and ratiometric fluorescence. The research on fluorescent probes is mostly based on the change in the emission intensity from a single wavelength, such as fluorescence quenching or enhancing probes [20,21]. Single-emission fluorescent probes are affected by photobleaching, probe concentration, minor changes in the environment, and illumination stability. In order to overcome the above shortcomings, there are increasing studies on ratiometric fluorescence methods. The basic principle of ratiometric fluorescence detection is to achieve ratiometric detection of analytes by calculating the ratio of two fluorescent signals, for which the fluorescence intensity of two different wavelength bands changes after the fluorescent probe is combined with the reactant. Compared with single-emission fluorescent probes, ratiometric fluorescent probes can significantly improve detection accuracy by avoiding detection errors caused by non-uniform probe concentrations, unstable excitation light sources, changes in the microenvironment of biological samples, photobleaching, the different parameter of cameras and unstable excitation light sources [22,23,24,25]. In short, ratiometric fluorescence sensors have received increasing research attention due to their simple operation, safety, good stability, and suitability for high-throughput screening applications [26]. In the past few years, the ratiometric fluorescent sensors have been extensively used in the detection of diverse analytes including metal ions [27,28,29], anions [30,31], organic pollutants [32], and biomacromolecules [33]. However, a smartphone-based ratiometric fluorescent sensor for the detection of Ca^2+^ content in bovine serum, to the best of our knowledge, has not been developed. More importantly, the smartphone-based detection systems can overcome the shortcomings of the conventional colorimetric method, for example, the error caused by background noise and color recognition. Therefore, the combination of smartphone and fluorescent sensing systems is a more intelligent, sensitive, and accurate measurement for calcium ions detection.

**Figure 1:**
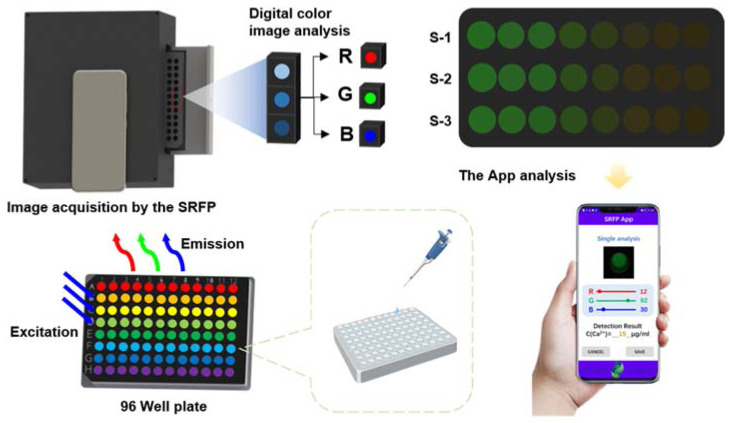
Schematic illustration of SRFP system and its application in bovine blood calcium sensing and concentration detection. A series of Cal Red R525/650 mixed solutions containing different Ca^2+^ ions content in bovine serum was put into a 96-well plate. Fluorescence images of samples at different known concentrations were obtained with the SRFP platform. The prediction of Ca^2+^ concentration in unknown bovine serum samples is achieved by converting the corresponding image information into the ratio of red and green channels through the built-in image processing program.

In this work, we propose a novel portable smartphone-based platform combined with ratiometric fluorescent probes for the detection of blood calcium (shown in Figure 1). For accurate quantitative analysis, we recorded the color change with a smartphone and converted the corresponding image information into the signal ratio between green and red channels through the image processing program of the self-made App, which showed a linear relationship with the calcium ion concentration. The signal readout from the designed application provided a feasible means of quantitative detection. More importantly, the test results obtained by our smartphone reader are comparable to those obtained by conventional fluorescence spectroscopy. The platform has the advantages of being inexpensive, easy portability, handleability, high throughput, good selectivity, and repeatability, etc., which provides a broad prospect for the practical application of POCT.

## 2. Experimental Section

### 2.1. Chemical and Reagents

Fluorescein isothiocyanate (FITC) was obtained from Shanghai Macklin Biochemical Technology Co., Ltd. (Shanghai, China) Cal Red R525/650, a 488 nm-excitable ratiometric fluorescence calcium indicator produced by AAT Bioquest Inc. (Sunnyvale, CA, USA), was purchased from Xi’an Biolite Biotech Co., Ltd. (Xi’an, China). The calcium ion composition analysis standard material in water (1000 ppm) was provided by Sichuan National Testing Technology Reference Materials Technology Co., Ltd. (Guanghan, China) Tris-HCl buffer (1 M, pH 7.4, Sterile, DNase free) was obtained from Shanghai Beyotime Biotechnology Co., Ltd. (Shanghai, China). Analytical grade chlorides (Cupric ion, Ferric ion, Potassium ion, Magnesium ion, Sodium, Zine ion) that did not require further purification were purchased from Shanghai Macklin Biochemical Technology Co., Ltd. Fetal Bovine Serum (FBS) was purchased from Biological Industries (Haifa, Israel). Ultrapure water used in the study was produced by a Millipore Milli-Q System (Millipore, Burlington, MA, USA). The materials used in the experiments were purchased from standard companies, and the solutions used were all prepared by conventional laboratory methods.

### 2.2. Platform Design and Assembly

**Figure 2:**
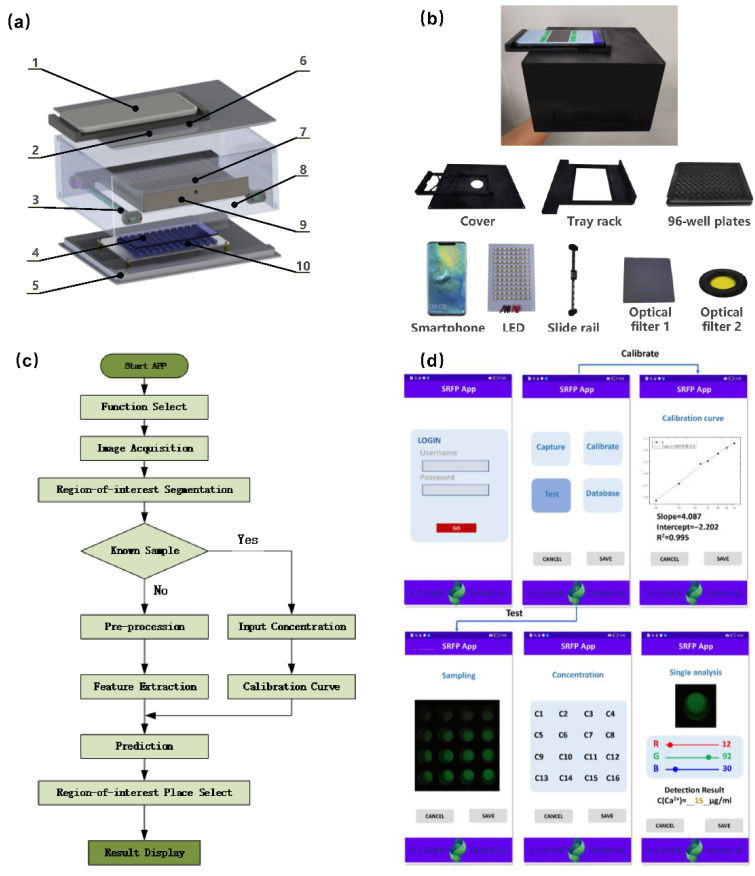
Schematic illustration of homemade SRFP platform and the self-made SRFP-App. (**a**) Details design of the SRFP platform are displayed. (**b**) Fluorescence responses were obtained from red, green, and blue channels at the same fluorescence intensity and exposure time. (**c**) Diagram showing the operation processing steps of the self-made SRFP-App. (**d**) Detailed operations of the SRFP-App software interface.

To achieve the practical application of POCT in bovine serum, we designed and assembled a low-cost, high-throughput, portable SRFP platform. As illustrated in Figure 2a, the SRFP platform consists of four main parts: a low-cost LED light source, a black box with a 96-well plate tray rack, optical filters, and smartphone. The specific information of each part is listed as follows: (1) A Mate 20 Pro smartphone (HUAWEI, China, 6.39 inches, 1440 × 3120 pixels) with high camera resolution used for image acquisition and data processing and analysis. (2) A top cover used to hold the smartphone in place and the optical filter 1. (3) Two slide rails which are connected to the black box. (4) Optical filter 1 (Narrowband filter, 460~490 nm, Geng Xu Optoelectronics Co., Ltd., Shenzhen, China) used to effectively filter out the LED light source except for the wavelength at 475 nm. (5) Bottom plate. (6) Optical filter 2 (Long-pass Filter, from 500 nm, Edmund Optics Co., Ltd., Shenzhen, China) used to prevent light below 500 nm from passing through. (7) The 96-well plate made of black polystyrene (for high-throughput detection) with a clear bottom and a black inner wall to hold the sample solution. (8) The black box body in the experiment which made of inexpensive flexible polystyrene (EPS) by 3D printing technology, and the inner and outer walls are treated as black light-absorbing surfaces without reflection effects to obtain a homogeneous interior environment. (9) The tray rack is used to place a 96-well plate and move on slide rails. (10) A total of 96 LEDs (100 mW in power) with a wavelength of 475 nm serve as the light source of excitation was controlled by a constant current module and powered by a common 12 V AA size lithium battery and the fluorescent images are collected by the smartphone camera. The entire device was designed by SolidWorks (Dassault Systems S.A, Paris, France), using EP epoxy resin as the printing material, and fabricated using a commercial 3D printer (Formlabs 3L, FORM LABS INC, Somerville, MA, USA). In addition, to eliminate the interference of ambient light and reduce specular reflection, black paint was sprayed on the inner wall of the dark box and the surface of the 96-well plate tray. Details of the SRFP platform and the overall structure are displayed in Figure 2b.

### 2.3. Android-Based Applications Development 

It is necessary to develop a color recognizer App based on the smartphone to obtain the RGB values corresponding to the fluorescent photos for further statistical analysis. Samples solution was placed in a 96-well plate, and the images were acquired in a homemade SRFP platform and calculated by the smartphone App. The smartphone App, called Blood Calcium Analyzer, was built with Android Studio (Google, Mountain View, CA, USA) for smartphone digital-image cropping, RGB color values extraction, and data calculation (Figure 2c). The concentration of analyte in the samples can be determined instantly by the smartphone. The image processing here uses an 8-bit color scale expression (values from 0 to 255, where white corresponds to a color intensity of 255 and black corresponds to 0). 

As shown in Figure 2c, this App mainly includes three aspects of functions: calibration curve, actual sample test with concentration prediction, and results display. For the first part, fluorescence images of samples at different known concentrations were obtained with the SRFP platform to serve as the standard calibration curve. For the second part, the fluorescence images of bovine serum with unknown concentrations of Ca^2+^ were captured, and a simple algorithm is applied in this process to identify the bright spot in the center of the image and the average value of nearby pixels. For the last part, we can choose to display the concentration of all samples or choose to display the details of the concentration of a single sample according to our own needs. The detailed operations of the SRFP-App software interface are shown in Figure 2d.

### 2.4. Detection for Ca^2+^ Based on Cal Red R525/650 Fluorescent Probe 

The analysis was performed in Tris-HCl buffer according to the following procedure, and data were collected at room temperature. Briefly, Cal Red R525/650 (25 μg/mL) and various concentrations of Ca^2+^ (0, 1, 2, 6, 10, 15, 18, 22, 25, 28, 30, 35, 40, 80, 150, and 250 μM) were combined in 100 μL and added to a 96-well plate. After incubation in the dark for 5 min, a series of fluorescence spectra and fluorescence images were recorded using a fluorescence spectrometer and the SRFP platform under excitation at 475 nm, respectively. For the fluorescence images, a smartphone in the SRFR platform was used to identify and convert colors into different RGB values, and the G/R values correlated with the quantitative detection of Ca^2+^.

### 2.5. Quantitative Detection of Ca^2+^ in Bovine Serum 

To evaluate the utility of the SRFP platform to detect bovine serum, we selected fetal bovine serum as a real sample for the detection of Ca^2+^. The fetal bovine serum was diluted 50 times with Tris-HCl buffer, and then five different concentrations of Ca^2+^ were added to the diluted serum as the test sample. Samples were examined similarly to the description in Section 2.4, a series of fluorescence spectra and fluorescence images were recorded, and spiked recoveries were analyzed.

### 2.6. Statistical Analysis

In this work, all experiments were performed three times and the data processing was expressed as mean ± standard deviation (SD). The Student’s *t*-test was used for the statistical evaluation of the data results. Treatment of the data and statistical analysis (average value and recovery) were performed using datasheets prepared in Microsoft Excel 2019 (Microsoft, Redmond, WA, USA) and all the drawings are made using the Origin 2018 drawing software (Origin Lab, Northampton, MA, USA).

## 3. Results and Discussion

### 3.1. Performance Test and Optimization of the SRFP Platform

**Figure 3:**
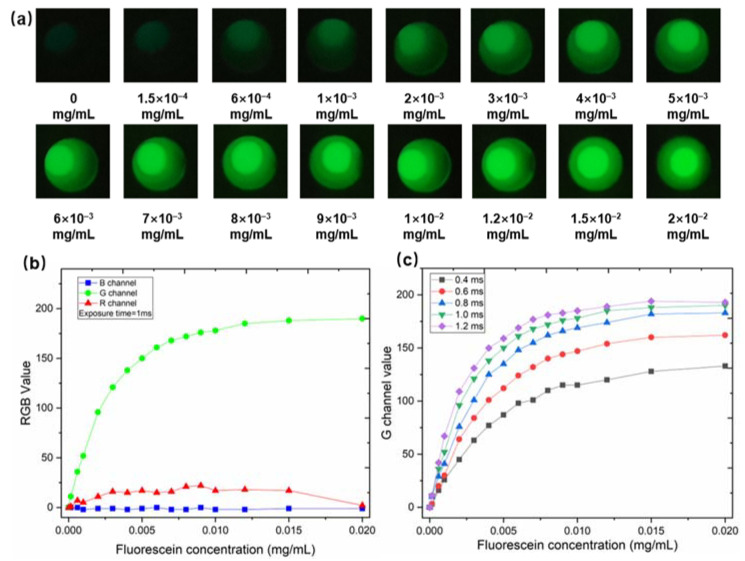
The result of fluorometric measurements using FITC as a standard model analyte. (**a**) The fluorescence images of the different concentrations of FITC solutions (between 0 and 2 × 10^−2^ mg/mL). (**b**) Fluorescence responses were obtained from red, green, and blue channels at the same excitation light intensity and exposure time. (**c**) Fluorescence responses of the green channel of fluorescein at the same excitation light intensity for different exposure times.

To verify the feasibility of the constructed SRFP platform, we selected a standard fluorescent solution (FITC) as the model analyte. FITC exhibits strong fluorescence, emitting green light at 525 nm after excitation at 488 nm. We prepared standard solutions with 0.1 M sodium hydroxide (NaOH) and diluted them due to their stronger fluorescence properties in alkaline solutions. Then, the 16 different sample solutions were placed in a 96-well plate, and images were acquired in the SERF platform. LEDs were controlled by a constant current module and powered by a common 12 V AA size lithium battery. The exposure time of the camera is set to 1 ms. Between 0 and 2 × 10^−2^ mg/mL, the captured fluorescence images of the different concentrations for FITC solutions are displayed in Figure 3a.

As can be observed from Figure 3b, while the fluorescence responses of the other channels (red and blue) were almost unchanged with the change in FITC concentration during the experiment, only the change in the intensity of the green channel was most consistent with the change in the concentration of FITC. In addition, appropriate exposure time is crucial for the SRFP platform. The effect of different exposure times (the exposure time is set as 0.4 ms, 0.6 ms, 0.8 ms, 1.0 ms, and 1.2 ms, respectively) on the performance of standard fluorescent probes is shown in Figure 3c. As the exposure time increases, the response of the green channel also increases correspondingly. When the concentration is 0.015 mg/mL and the integration time exceeds 1 ms, the value of the G channel reaches saturation. To obtain the maximum dynamic response under the same exposure time, we chose 1 ms as the optimal exposure time for the SRFP platform.

### 3.2. Detection for Ca^2+^ Based on Cal Red R525/650 by Fluorescence Spectra

Ratiometric fluorescent probes with dual-emission fluorescence properties have a self-calibration function, which can improve the stability and accuracy of fluorescent sensors [22,23]. In this work, we selected Cal Red R525/650 as the ratiometric fluorescent probes. Cal Red R525/650 is composed of calcium chelator (ligand) and fluorescent chromophore group in molecular structure. Ca^2+^ in serum will be specifically coupled by the ligand in Cal Red R525/650, resulting in changes in molecular conformation and spectral properties of fluorescent chromophore group. When Cal Red R525/650 is excited at 488 nm, the transmitted signal increases at 519 nm and decreases at 665 nm [34]. To determine the optimum concentration range of the fluorescent probe, a series of Cal Red R525/650 mixed solutions containing different concentrations of Ca^2+^ was put into a 96-well plate and the fluorescence spectra of these solutions were detected using a fluorescence spectrometer.

**Figure 4:**
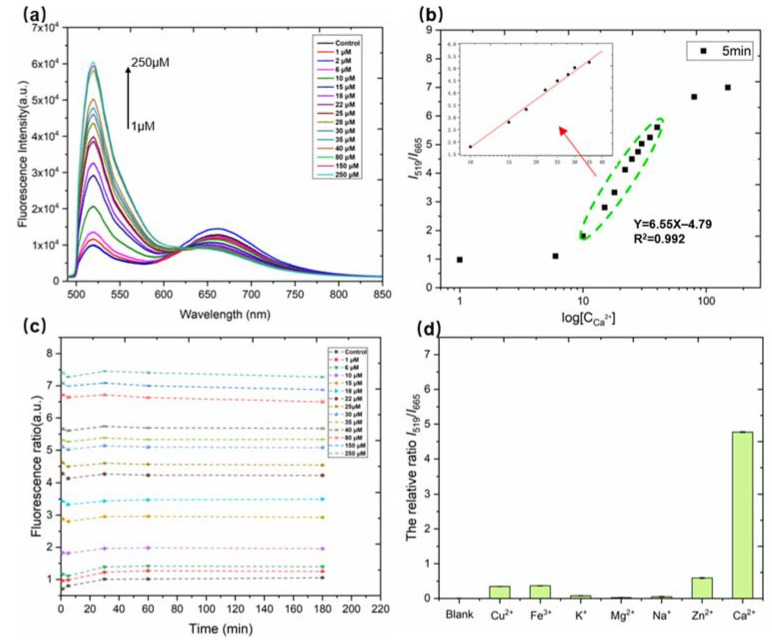
(**a**) Fluorescence spectra of the ratiometric probe (Cal Red R525/650) solution when different concentrations of Ca^2+^ were added. The concentrations of Ca^2+^ from bottom to top are 0, 1, 2, 6, 10, 15, 18, 22, 25, 28, 30, 35, 40, 80, 150, and 250 μM, respectively. (**b**) The relationship between the fluorescence intensity ratio (I_519_/I_655_) and different concentrations of Ca^2+^ (log [Ca^2+^]). (**c**) Time-dependent fluorescence intensity ratio I_519_/I_655_ of the ratiometric probe (between 0 and 180 min). (**d**) Selectivity tests for the probe were conducted. Error bar indicates mean ± SD for three measurements at different places.

The relationship between fluorescence intensity and calcium ion concentration (log [Ca^2+^]) is discussed below. Under the optimal detection conditions, the fluorescence spectra of ratiometric probe solution toward Ca^2+^ of various concentrations (0, 1, 2, 6, 10, 15, 18, 22, 25, 28, 30, 35, 40, 80, 150, and 250 μM) were determined (as illustrated in Figure 4a). The fluorescence intensity at 665 nm decreased with the strengthen of the fluorescence intensity at 519 nm. To construct a sensing system with stable signal output, the ratio of the fluorescence intensity at 519 nm to the fluorescence intensity at 665 nm (I_519_/I_665_) was set to be the readout value. The fluorescence intensity ratio I_519_/I_665_ was linearly correlated with different Ca^2+^ concentrations in the range from 10 μM to 40 μM with a decent correlation coefficient of 0.993 (as illustrated in Figure 4b).

Stability is an important indicator to measure the detection capability of the ratiometric fluorescent probe. The changes in the fluorescence ratio of the probes (Cal Red R525/650) with different calcium ion concentrations with time (0~180 min) were studied. As shown in Figure 4c, the fluorescence ratio I_519_/I_665_ was plotted as a function of incubation time, where I_519_/I_665_ represented the fluorescence intensity ratio at 519 nm and 665 nm after adding different concentrations of Ca^2+^ to the fluorescent probe at the same time interval. As the concentration of calcium increased, the fluorescence intensity ratio I_519_/I_665_ at the same time point also increased continuously, reaching the maximum value at about 250 μM (Figure 4c). However, the fluorescence intensity ratio I_519_/I_665_ hardly changed with time at the same calcium concentration. Therefore, the probe has very good stability and can be suitable for the following experiments.

Selectivity is another crucial metric to assess the ability in the ratiometric fluorescent probe. The calcium sensitive domain of Cal Red R525/650 is the calcium chelator, which may bind to other metal ions with similar chemical properties in serum. Thus, possible potential interference with calcium detection by other interfering ions was explored. Solutions containing Ca^2+^ and different kinds of interfering ions (including Mg^2+^, Zn^2+^, Cu^2+^, Na^+^, K^+^, and Fe^3+^) were pretreated according to the recommended procedure. Under the same conditions, the response of Ca^2+^ and possible interfering analytes to the fluorescence intensity ratio I_519_/I_655_ was determined at a concentration of 25 μM and data were normalized in processing. As shown in Figure 4d, the fluorescence ratio I_519_/I_655_ value changed significantly in the presence of Ca^2+^, whereas the fluorescence ratio I_519_/I_655_ values barely changed in the presence of other interfering ions. In addition, the concentrations of these metal ions were Mg^2+^ (~0.02 mM), Zn^2+^ (0.001 mM), Cu^2+^ (0.001 mM), Fe^3+^ (0.001 mM), Na^+^ (3 mM), K^+^ (0.1 mM), far exceeding their concentrations in bovine serum (after 50-fold dilution) [35]. Therefore, the competitive experiment of the Ca^2+^ detection in the presence of interference ions demonstrated that the ratiometric probe solution has a good selectivity toward Ca^2+^ detection.

### 3.3. Quantitative Detection of Calcium-Based SRFP

**Figure 5:**
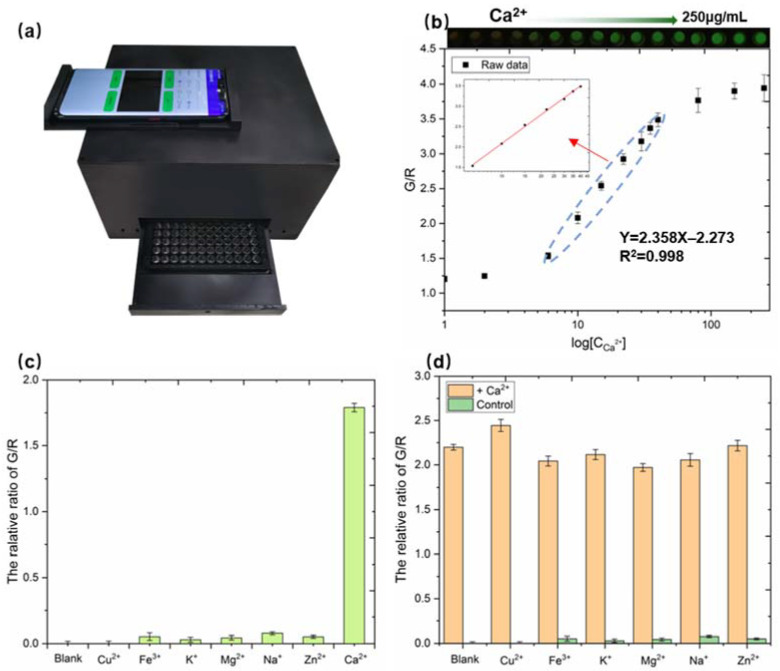
(**a**) The samples of different Ca^2+^ concentrations were tested by the SRFP platform. (**b**) The relationship results between the G/B value and the different concentrations of Ca^2+^. (**c**) Selectivity of the fluorescence ratiometric probes for detecting Ca^2+^ (25 μM) and interfering substances (Cu^2+^, Fe^3+^, K^+^, Mg^2+^, Na^+^, Zn^2+^). (**d**) The relative value ratio is Green /Red of the mixed with different interfering substances (Cu^2+^, Fe^3+^, K^+^, Mg^2+^, Na^+^, Zn^2+^). Tests were performed in triplicate. Error bar indicates mean ± standard deviation for three measurements at different places.

To determine the effective range of calcium concentrations for blood serum detection, the samples of different Ca^2+^ concentrations were tested by the SRFP platform (shown in Figure 5a). We selected the green channel and the red channel, and the signal ratio of the green channel to the red channel was plotted versus the logarithm of different Ca^2+^ concentrations (log [Ca^2+^]). As shown in Figure 5b, the G/B value has a linear relationship with the concentration of Ca^2+^. The linear equation in the range of 6~40 μM is y = 2.358x − 2.273 and R^2^ = 0.998. According to the methods recommended by the International Union of Pure and Applied Chemistry (IUPAC), 10 blank samples (without Ca^2+^) were tested in Tris-HCl buffer. The LOD was calculated to be 1.8 μM according to the following formula: 3σ/k (of which σ denotes the standard deviation, and k is the slope of the calibration curve). Therefore, we prove here that the portable quantitative detection of Ca^2+^ can be achieved using just a smartphone.

Excellent selectivity to shield from interference are a necessary capacity for a ratiometric fluorescence sensing system. To demonstrate the selectivity of the developed SRFP platform system, a number of potential substances were measured under the same conditions and data were normalized. As shown in Figure 5c, the signal ratio of the green channel to the red channel in the fluorescence image of the probe solution hardly changed in the presence of these competitors. Subsequently, the value ratio of the G/R channel was rapidly increased after adding 25 μM Ca^2+^ to the fluorescence ratiometric probe solution. The fluorescence response value of Ca^2+^ was about 40 times higher than that of other metal ions.

To discuss the specificity of ratiometric fluorescence probes for Ca^2+^ detection in more detail, we performed another perturbation experiment when the presence of other interferences with Ca^2+^. In the solution in the presence of different interfering ions, Ca^2+^ of the same concentration was added sequentially. As shown in Figure 5d, the specific fluorescence intensity of the SRFP platform changed significantly with the addition of 25 μM Ca^2+^. At the same time, the other interferences used above did not produce such a significant change in ratiometric fluorescence change in comparison. This phenomenon manifested that the SRFP sensing system has excellent anti-interference for Ca^2+^, which indicates the wide applications of such system in complex samples. We also compared the SRFP performance with other reported sensors for Ca^2+^ determination (shown in Table 1). Although the linear range of SRFP was smaller than the other reported sensors, the detection limit was better than the others. In addition, the SRFP platform can detected quickly for bovine blood calcium with simple operation.

### 3.4. Application in the Detection of Calcium in Serum-Based SRFP

**Figure 6:**
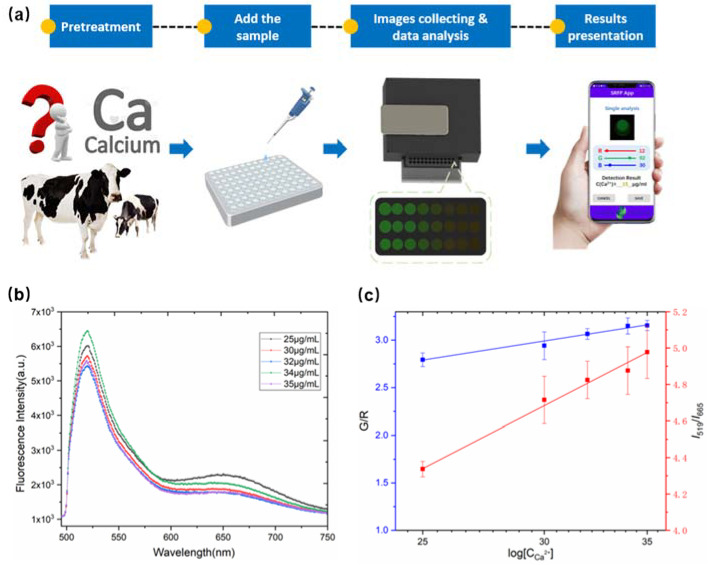
(**a**) Schematic illustration of the SRFP system and the application of detection of Ca^2+^ in bovine serum. (**b**) Fluorescence spectra of bovine serum with different levels of Ca^2+^ with ratiometric fluorescent probes. (**c**) A linear relationship between the ratio of G/R channel (blue line) and the fluorescence intensity ratio I_519_ /I_665_ (red line) and different concentrations of Ca^2+^. Tests were performed in triplicate. Error bar indicates mean ± standard deviation for three measurements at different places.

To further evaluate the application of the SRFP platform in actual sample detection, our sensing platform was applied to detect blood calcium. Figure 6a shows the process of visual and quantitative detection of Ca^2+^ in bovine serum samples through the SRFP platform. The specific sample testing steps are as follows: Firstly, five different concentrations of Ca^2+^ (0, 5, 7, 9, and 10 μM) were added to serum diluted 50-fold with Tris-HCl as test samples. Then, the ratio probe is put into the samples in a 96-well plate. Subsequently, the processed samples are placed in the SRFP platform, and a smartphone is used for fluorescence image capture and concentration analysis. Meanwhile, the fluorescence spectrum is measured and recorded by a fluorescence spectrophotometer, and the results are shown in Figure 6b. Next, the Blood Calcium Analyzer App converts the acquired fluorescence images to numeric values representing the R, G, and B color modes. Finally, the fluorescence image of interest is captured and the ratio of green and red (G/R) channels is calculated for quantitative analysis of Ca^2+^. We obtained a favorable linear relationship between the ratio of G/R channel (blue line) and the concentrations of Ca^2+^ (shown in Figure 6c). The linear equation in the range of 20~40 μM is y = 2.358x − 2.273 and R^2^ = 0.998.

Moreover, the data analyzed by the homemade App of the smartphone were compared with the data detected by fluorescence spectra detection. The results of the data show that there was no statistically significant difference in the assay between the method using fluorescence spectra detection and the method proposed in this work. Recovery experiments were performed to test all bovine serum sample varieties by adding five levels of Ca^2+^ component solutions. The measurement results are shown in Table 2, the recoveries of real samples ranged from 92.8% to 110.1%, with RSD (*n* = 3) ranging from 1.72% to 4.89%. To further verify the accuracy of the method, we also detected the calcium ion concentration in these serum samples by fluorescence spectroscopy. The detection results of the SRFP platform are consistent with the detection results of fluorescence spectroscopy, which proves that the SRFP platform has good accuracy and reliability.

The results demonstrate that the smartphone-based platform can detect different concentrations of Ca^2+^ in bovine serum with high accuracy and cost-effective. It is obvious that a better linear relationship between concentration and intensity is obtained on our SRFP platform. Moreover, the method allows better quantitative analysis compared to fluorescence spectroscopy. 

## 4. Conclusions

In conclusion, we propose a novel sensing platform for Ca^2+^ concentrations detection in bovine serum by employing a smartphone-based ratiometric fluorescent probe to achieve early warning and initial diagnosis of hypocalcemia. Under the optimal conditions, the anti-interference and selectivity performance of the smartphone-based ratiometric fluorescent sensor for Ca^2+^ detection is excellent in presenting other interfering ions. Utilizing the developed smartphone-based platform, a clear fluorescent color change can be observed when the Ca^2+^ concentration level is abnormal. Quantitative detection of Ca^2+^ was achieved with a minimum detectable concentration of 1.8 μM in bovine serum samples. The test results of real samples demonstrate the feasibility and sensitivity of the smartphone-based ratiometric fluorescence sensing platform for Ca^2+^ concentration detection. The designed ratiometric fluorescent probe-based sensing platform exhibit good sensitivity and practicality, proves great potential in the early screening detection of hypocalcemia, and further accelerate the development of the real-time/on-site detection equipment research field. Moreover, due to the specific luminescence properties of the analyte, this setup can be extended to different fluorescence detection applications, such as water quality testing, and food processing.

## Figures and Tables

**Table 1:** Comparison of SRFP performance with other reported sensors for Ca^2+^ determination.

Sensor	Method	Linear Range (M)	Detection Limit (M)	Ref
GCE/SWNT/DNA zyme	EIS	5 × 10^−6^~2.5 × 10^−6^	4.2 × 10^−6^	[36]
GCE/NGR/AuNPs/Sub-DNAzyme	EIS	5 × 10^−6^~5 × 10^−5^ 5 × 10^−5^~4 × 10^−4^	3.8 × 10^−6^	[37]
CalTreAX	Fluorescence	1 × 10^−6^~1 × 10^−3^	1 × 10^−5^	[38]
SRFP	Ratiometric fluorescence	1 × 10^−5^~4 × 10^−5^	1.8 × 10^−6^	This work

**Table 2:** Comparison results of bovine serum calcium detection using fluorescence detection method and SRFP platform.

Detection Mode	Spiked Concentration (μg/mL)	Recovery (*n* = 3, %)	RSD (*n* = 3, %)
Fluorescence	0	-	-
	5	92.8	4.89
	7	102.4	1.83
	9	108.9	2.68
	10	99.4	1.72
SRFP platform	0	-	-
	5	115.7	4.86
	7	110.1	4.20
	9	96	2.68
	10	105.9	3.61

## Data Availability

Datasets generated during and/or analyzed during the current study are available from the corresponding author upon reasonable request.
